# The genetic risk of acute lymphoblastic leukemia and its implications for children of Latin American origin

**DOI:** 10.3389/fonc.2023.1299355

**Published:** 2024-01-09

**Authors:** Adam J. de Smith, Silvia Jiménez-Morales, Juan Manuel Mejía-Aranguré

**Affiliations:** ^1^ Center for Genetic Epidemiology, Department of Population and Public Health Sciences, University of Southern California Keck School of Medicine, Los Angeles, CA, United States; ^2^ USC Norris Comprehensive Cancer Center, University of Southern California Keck School of Medicine, Los Angeles, CA, United States; ^3^ Laboratorio de Innovación y Medicina de Precisión, Núcleo A, Instituto Nacional de Medicina Genómica, Ciudad de México, Mexico; ^4^ Laboratorio de Genómica Funcional del Cáncer, Instituto Nacional de Medicina Genómica, Ciudad de México, Mexico; ^5^ Facultad de Medicina, Universidad Nacional Autónoma de México, Ciudad de México, Mexico

**Keywords:** childhood acute lymphoblastic leukemia, ALL, genetics, disparities, Hispanic/Latino, Latin America, genetic epidemiology, single nucleotide polymorphisms

## Abstract

Acute lymphoblastic leukemia (ALL) is the most common cancer in children, and disproportionately affects children of Hispanic/Latino ethnicity in the United States, who have the highest incidence of disease compared with other racial/ethnic groups. Incidence of childhood ALL is similarly high in several Latin American countries, notably in Mexico, and of concern is the rising incidence of childhood ALL in some Hispanic/Latino populations that may further widen this disparity. Prior studies have implicated common germline genetic variants in the increased risk of ALL among Hispanic/Latino children. In this review, we describe the known disparities in ALL incidence as well as patient outcomes that disproportionately affect Hispanic/Latino children across the Americas, and we focus on the role of genetic variation as well as Indigenous American ancestry in the etiology of these disparities. Finally, we discuss future avenues of research to further our understanding of the causes of the disparities in ALL incidence and outcomes in children of Latin American origin, which will be required for future precision prevention efforts.

## Introduction

Acute lymphoblastic leukemia (ALL) is the most commonly occurring malignancy in children, with a peak age at diagnosis of 2 to 5 years of age, and it remains a leading cause of childhood mortality (1, 2). The etiologies of childhood ALL are multifactorial, with several established risk factors that have large effects on disease susceptibility but are uncommon in the population, such as ionizing radiation and genetic syndromes, as well as more common exposures with small to moderate effects including birth weight, male sex, and single nucleotide polymorphisms (SNPs) (3, 4). Thus, not all children have an equal likelihood of developing ALL; for example, those harboring pathogenic germline variants in ALL predisposition genes have a relatively high risk of disease (5, 6). Furthermore, individuals of self-reported Hispanic/Latino ethnicity have the highest reported risk of developing ALL out of any population group in the United States (7, 8). We note that race/ethnicity groupings are socially constructed, and that Hispanics/Latinos are a highly heterogeneous group comprising individuals who originate from countries across the Americas and who are culturally, phenotypically, and genetically diverse. Nevertheless, the disparity in ALL incidence that disproportionately affects Hispanic/Latino children warrants investigation (9) – understanding the etiologies of childhood ALL and the causes of disparities in incidence will be essential for future disease prevention. In this review, we describe the disparities in ALL incidence and patient outcomes in children of Latin American origin and discuss the contribution of genetic variation and future directions of research.

## Disparities in ALL incidence in children of Latin American origin

### Hispanics/Latinos in the United States

**Figure 1:**
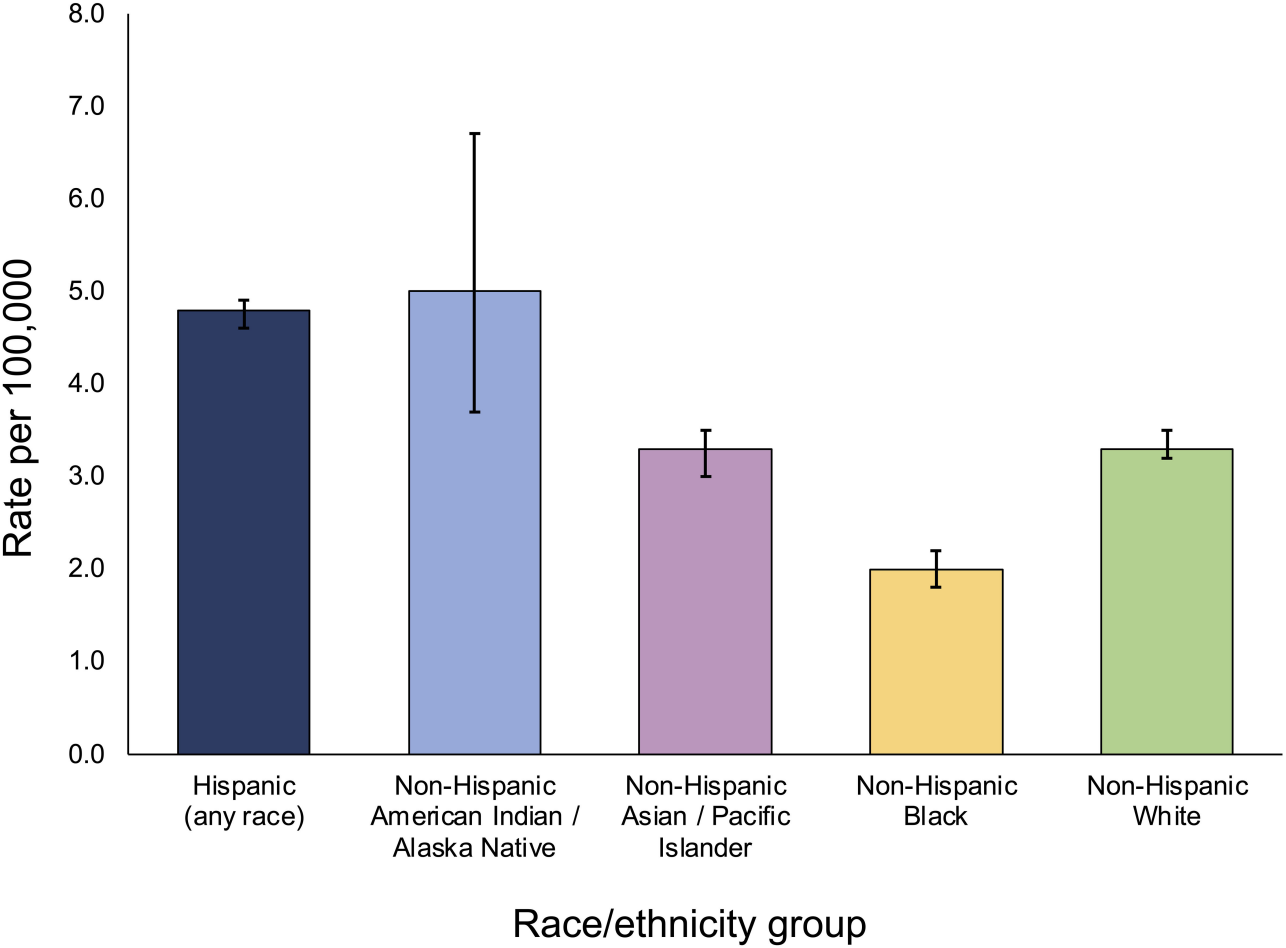
5-year age-adjusted incidence rates of childhood acute lymphoblastic leukemia across population groups in SEER, 2016-2020. Incidence rates are per 100,000 including both sexes and individuals < 20 years of age, and are age-adjusted to the 2000 US standard population. Bars represent 95% confidence intervals. Incidence data for Hispanics and non-Hispanics are based on the NAACCR Hispanic Latino Identification Algorithm. Rates for Non-Hispanic American Indian/Alaska Native include cases that are in a Purchased/Referred Care Delivery Area. Figure generated from data downloaded from the SEER*Explorer website (reference 16).

In the United States, there is a well-established racial/ethnic disparity in childhood ALL incidence rates, as reported in the National Cancer Institute’s Surveillance, Epidemiology, and End Results (SEER) program (**Figure 1**). Hispanic/Latino children have an approximately 30 to 40 percent increased risk of developing ALL than non-Hispanic White children, and a more than 2-fold higher risk than African American/Black children (8). Hispanics/Latinos are in general a genetically admixed population, with genetic ancestry largely derived from Indigenous American, European, and African populations (10). Thus, in the United States, individuals harboring greater proportions of Indigenous American ancestry in their genomes appear to have a higher risk of developing ALL, which may be related to differences in exposures to environmental risk factors but also suggests a role for genetic variation, as discussed later.

An association between lower socioeconomic status (SES) and an increased risk of childhood ALL was reported in Hispanics/Latinos in the United States using SEER data, which contrasted the positive relationships found in other racial/ethnic groups (11). Further analysis within each SES strata revealed that the difference in childhood ALL incidence between Hispanics/Latinos and non-Hispanic Whites was greatest in the lowest SES stratum and narrowed with increasing SES, becoming non-significant in the highest stratum (12). Given that Hispanics/Latinos with higher SES will on average harbor higher proportions of European ancestry (13), this suggested that the inverse relationship between SES and ALL risk may be confounded by Indigenous American ancestry and its potential association with an increased genetic risk for ALL and possibly with environmental risk factors (14).

The significant disparity in ALL incidence in the United States persists across the lifespan, with Hispanic/Latino adolescents and young adults (AYAs) as well as older adults having an approximately 2-fold greater risk of ALL than their non-Hispanic White counterparts (8). Furthermore, assessment of the trends of SEER incidence rates over time have revealed that the age-adjusted incidence rate of ALL in all age groups has been increasing significantly over recent decades in Hispanics/Latinos but not in non-Hispanic Whites (8, 15), with annual percent changes of ~0.64% in Hispanic/Latino children and ~2.0% in AYAs from 2000 to 2016. The causes of the rising incidence rates of ALL are unknown, but it suggests that the disparity in ALL incidence between Hispanics/Latinos and other population groups may increase further in the coming years. We do note that the most recent data in SEER (16) show that the incidence of ALL in Hispanics/Latinos appears to have stabilized in children since 2016, although it is still rising in AYAs, and it will be important to monitor these trends moving forwards.

### Hispanics/Latinos in Latin America

Outside of the United States, some of the highest global incidences of childhood ALL have been reported in Mexico and Costa Rica (17–19). In Mexico City, for example, the most recently estimated age-adjusted incidence rate of ALL in children aged 0-14 years was 5.3 per 100,000 (20), which is similar to the 5.1 per 100,000 rate reported in Hispanic/Latino children of the same age group in the United States (8). Among the municipalities in Mexico City, the highest incidence rates of ALL were reported in Iztacalco (6.9 per 100,000), Venustiano Carranza (6.7 per 100,000), and Benito Juárez (6.3 per 100,000), which are characterized by a low SES. This pattern may reflect an association between Indigenous American ancestry and ALL risk, given that poverty rates are higher among Indigenous people in Mexico (21, 22), but also highlights a potential role for environmental exposures linked to lower SES, such as air pollution, severe infection, and low-frequency magnetic fields (23, 24).

In South America, the highest incidence rates of ALL among countries with available data were reported in Ecuador and Colombia, countries similar to Mexico in that their populations are predominantly of mixed European and Indigenous American ancestry (“mestizo”), whereas the lowest incidence was reported in Argentina, a country where the population is largely European in its origins (19). Furthermore, in Brazil there is remarkable variation in ALL incidences between different regions in the country itself. The highest age-adjusted incidence rate (5.7 per 100,000) has been reported in Manaus, a city in the Amazonas region in which the population is highly indigenous, whereas lower incidences (2.2 to 4.6 per 100,000) are reported in regions along the Atlantic coast where the Brazilian population has greater contributions of European and African ancestry (19, 25). It is interesting to note that in the United States, non-Hispanic American Indian/Alaskan Native children have the highest incidence of ALL, at 5.0 per 100,000 individuals, although this is based on a small sample size available in SEER data (**Figure 1**). Together, these patterns in ALL incidences across the Americas suggest a possible association between Indigenous American ancestry and childhood ALL risk, which is supported by results from genetic studies as discussed below.

It has recently been reported that the incidence of ALL in Hispanic/Latino children in Puerto Rico is lower than in the rest of the Hispanic/Latino population in the United States, even with an incidence similar to that reported in non-Hispanic Whites (26). However, the childhood ALL incidence in Puerto Rico has been increasing significantly over recent years, at an annual percent change of almost 5%, although the causes of which remain to be determined (26). This may be analogous to the accelerated increase in ALL incidence in Mexico City in the 1980s (27), whereby the incidence of ALL was initially even lower than the average incidence in the United States but eventually overtook and subsequently exceeded it by almost 40%. It is possible that this may be occurring in the population of Puerto Rico and that in the near future the incidence of childhood ALL may reach a level higher than that of non-Hispanic Whites and similar to that of Hispanics/Latinos in the United States (28).

Considering these trends in Mexico City in the 1980s and currently in Puerto Rico, the high incidence rate of childhood ALL among Hispanics/Latinos may be a relatively recent phenomenon (28). It is possible that the susceptibility of Hispanics/Latinos to ALL has historically been relatively high but more recent exposures to physical, chemical, or biological factors may have led to an increase in ALL prevalence over time. In this review, we present evidence from studies of genetic variation in childhood ALL which, in addition to the aforementioned observations from cancer incidence data, suggest that Hispanics/Latinos are more susceptible to developing ALL than other racial/ethnic groups.

## Disparities in survival of childhood ALL patients of Latin American origin

### Inferior survival of Hispanic/Latino ALL patients in the United States and Latin America

The inferior survival and outcomes of Hispanic/Latino childhood ALL patients in the United States have been known for decades (29–31) and persist despite improvements in patient outcomes over time (32). In Latin American countries, even though ALL treatment is based on chemotherapy schemes used in high income countries (HICs), little improvement in overall survival (OS) and disease-free survival rates in pediatric ALL patients has been observed. Regarding event-free survival (EFS), during the last two decades 5-year survival rates for pediatric ALL patients in Mexico have been reported at between ~52 to 62% (33–35). Similarly low EFS has been reported for childhood ALL patients in El Salvador and Guatemala, with EFS of only 48% and 56%, respectively (36, 37). In a multinational study that included children (0-14 years) with ALL diagnosed during 2010-2014 from 61 countries, 5-year net survival rates were reported to be >80% in HIC, reaching more than 95% in Finland and Qatar (38). In contrast, Latin American countries displayed a wide range of lower survival rates with, for example, the lowest survival rate in Ecuador (50%), followed by Mexico (53%), and Peru (60%). Puerto Rico was the only region in Latin America showing a similar survival rate to many HICs (93%) (38, 39).

Further, and in contrast to HICs, high mortality rates persist for Hispanic/Latino pediatric patients with ALL. Some of the highest mortality rates have been reported in Mexico and Ecuador (40) and unfortunately, mortality rates in Mexican children with ALL were recently reported to have increased between 1998 and 2018 (41). Indeed, the age-adjusted mortality rates in childhood ALL patients in Mexico were reported to be 2.5-fold higher than the mortality rates of Mexican American patients in California (born in the United States but of Mexican origin) (42).

### Indigenous American ancestry and ALL patient outcomes

Higher rates of relapse in Hispanic/Latino ALL patients in the United States and in Latin American countries contribute to their inferior OS and EFS (43–45). The causes of the differential survival rates of ALL patients among Hispanic/Latino populations are, however, complex and multifactorial. It is well known that superior rates of OS are influenced by more effective risk-directed therapies, implementation of supportive strategies to overcome chemotherapy toxicity, accurate diagnostic tools, targeted treatment base on the genomic background, more accessibility to bone marrow transplantation, better access to care, and treatment adherence (45–47). SES has been shown to significantly mediate the survival disparity between Hispanic/Latino and non-Hispanic White ALL patients, although the disparity remains after accounting for SES (48). There is evidence that genetic ancestry can influence treatment response and survival of ALL patients in Hispanic/Latino populations (45). Furthermore, in a population-based study in the United States, Shoag et al. found that mortality rates in “Continental” Hispanic/Latino childhood ALL patients, *i.e.* those originating from Mexico, Central America, or South America, were approximately two-fold higher than the mortality rates in Hispanic/Latino patients of Caribbean (Puerto Rico, Cuba, Dominican Republic) origin (49). In studies considering patient genomic characteristics, it has been reported that approximately two-thirds of the ancestry-related differences in EFS may be explained by ALL molecular subtypes (50). In addition to having a lower frequency of good prognostic molecular subtypes including *ETV6::RUNX1* fusion gene and high hyperdiploidy, childhood ALL patients with more Indigenous American ancestry had a higher frequency of the poor prognosis molecular subtypes, such as *CRLF2* rearrangements and Philadelphia chromosome-like (Ph-like) ALL (50). A similar finding was reported by Gupta et al. who found that after controlling for treatment regimen and insurance type, Hispanic/Latino children with B-cell ALL still had shorter survival than children of other race/ethnicities; however, these differences by race/ethnicity were not found in children with T-cell ALL (32). Furthermore, Lee et al. (50) reported an association between Indigenous American ancestry and poor prognosis, even with contemporary ALL therapy and after adjusting for genomic and clinical features, suggesting additional factors contribute to this disparity.

Latin America is a large region comprising South America, Central America, Mexico, and the Caribbean territories made up of 26 countries, and Latin American populations are ancestrally heterogeneous originating from ancient and dynamic migration processes of Northeastern Asia into America, the European colonization beginning in the 16^th^ century, and the transatlantic slave trade from West Africa (51). Considering the findings showing an association between Indigenous American ancestry and poor survival in Hispanic/Latino patients, it is notable that populations in Guatemala (55%), Mexico (62%), Ecuador (51%), and Peru (50%) have the highest proportions of Indigenous American genetic ancestry compared with other Latin American populations (52–54). Given the high mortality rates of ALL among Latin American populations, and that genetic ancestry has been shown to contribute to the variance in EFS independent of molecular subtypes (50), consideration of ancestry in addition to molecular subtype information may improve outcomes of ALL patients and contribute to the establishment of evidence-based health policies. Further, to alleviate this disparity in children’s health, it is essential to gain a full understanding of the etiologies underlying the increased risk of childhood ALL in children of Latin American origin. Below, we summarize the current knowledge on the role of genetic variation in childhood ALL risk in Hispanics/Latinos, largely based on studies performed in the United States, and the implications for children in Latin America.

## Genetic variation and ALL risk in Hispanic/Latino children in the United States

Genome-wide association studies (GWAS) have established a genetic contribution to the development of childhood ALL, with common variants identified in at least a dozen well-replicated risk loci (**Table 1**) (55–67). Several of the implicated ALL risk genes, including *IKZF1*, *ARID5B*, *CEBPE*, *GATA3*, and *ERG*, encode transcription factors that are involved in lymphocyte development and hematopoiesis, suggesting that disruption of blood cell regulation and immune function are involved in the etiology of childhood ALL. Several ALL-associated SNPs, or variants in nearby genomic regions, have also been associated with variation in blood cell phenotypes (60, 61, 68). Moreover, in a recent Mendelian randomization study of blood cell traits in childhood ALL, it was demonstrated that a genetic predisposition to overproduction of lymphocytes is associated with an increased ALL risk, albeit this study was limited to individuals of European ancestry (69).

**Table 1:** Childhood acute lymphoblastic leukemia (ALL) GWAS association loci and their risk allele frequencies across populations.

Gene	Region	SNP *	Ref	Alt	Risk	ALL subtype association	PubMed ID	First author (year)	*P-*value	OR (95% CI)	RAF in AFR	RAF in AMR **	RAF in EUR	RAF in HGDP Native Americans ***
*BCL11A*	2p16.1	rs2665658	C	A	A	*TCF3-PBX1* ALL	32882024	Lee SHR (2021)	1.88E-08	4.00 (2.47 to 6.49)	0.461	0.358	0.363	0.171
*RPL6P5*	2q22.3	rs17481869	C	A	A	B-cell ALL (*ETV6-RUNX1*)	29632299	Vijayakrishnan J (2018)	3.20E-08	2.14 (1.64-2.80)	0.022	0.039	0.081	0.000
*C5orf56*	5q31.1	rs886285	T	C	T	B-cell ALL (High-hyperdiploidy)	31767839	Vijayakrishnan J (2019)	1.56E-08	1.29 (1.18-1.41)	0.648	0.323	0.329	0.186
*BAK1*	6p21.31	rs210143	T	C	C	B-cell ALL (High-hyperdiploidy)	31767839	Vijayakrishnan J (2019)	2.21E-08	1.30 (1.19-1.43)	0.735	0.739	0.718	0.700
*MYB/HBS1L*	6q23	rs9376090	T	C	T	ALL	34750507	Jeon S (2021)	8.23E-09	1.27	0.957	**0.841**	0.743	0.900
*IKZF1*	7p12.2	rs10272724	G	A	A	ALL	19684604	Papaemmanuil E (2009)	1.00E-19	1.69 (1.58-1.81)	0.180	0.233	0.271	0.235
*IKZF1*	7p12.2	rs4917017	G	A	A	ALL	N/A	de Smith AJ (2023)	2.05E-17	1.41 (1.33-1.49)	0.283	**0.432**	0.295	**0.800**
*IKZF1*	7p12.2	rs76880433	C	T	T	ALL	N/A	de Smith AJ (2023)	4.67E-11	1.44 (1.33-1.55)	0.033	**0.176**	0.002	**0.443**
*CCDC26*	8q24.21	rs4617118	A	G	G	ALL	29348612	Wiemels JL (2018)	3.05E-09	1.27 (1.17-1.38)	0.299	0.147	0.174	0.043
*CDKN2A*	9p21.3	rs3731249 *	C	T	T	ALL	26527286	Walsh K (2015)	1.69E-13	2.97 (2.22-3.96)	0.005	0.026	0.031	0.000
*CDKN2A/B*	9p21.3	rs2811711	T	C	T	ALL	34750507	Jeon S (2021)	1.85E-11	1.36	0.841	0.889	0.861	**1.000**
*CDKN2B*	9p21.3	rs77728904	A	C	C	B-cell ALL	26868379	Hungate EA (2016)	3.32E-15	1.72 (1.50-1.97)	0.092	0.060	0.078	0.000
*TLE1*	9q21.31	rs76925697	A	T	A	B-cell ALL	31767839	Vijayakrishnan J (2019)	2.11E-08	1.52 (1.31-1.76)	0.962	0.967	0.963	**1.000**
*PIP4K2A*	10p12.2	rs7088318	C	A	A	ALL	23512250	Xu H (2013)	1.13E-11	1.40 (1.28-1.53)	0.395	**0.704**	0.603	**0.957**
*BMI1*	10p12.31	rs11591377 *	G	A	G	ALL	29923177	de Smith AJ (2018)	2.07E-10	1.27 (1.20-1.35)	0.907	0.772	0.789	0.643
*GATA3*	10p14	rs3824662 *	C	A	A	B-cell ALL (Ph-like)	24141364	Perez-Andreu V (2013)	2.17E-14	3.85 (2.71-5.47)	0.099	**0.294**	0.175	**0.543**
*ARID5B*	10q21.2	rs7090445 *	C	T	C	ALL	19684603	Treviño LR (2009)	1.40E-15	1.91 (1.60-2.20)	0.212	**0.441**	0.324	**0.671**
*JMJD1C*	10q21.3	rs9415680	A	G	A	ALL	34750507	Jeon S (2021)	7.27E-08	1.20	0.040	**0.292**	0.146	**0.714**
*TET1*	10q21.3	rs10998283	G	A	A	ALL	34750507	Jeon S (2021)	3.92E-08	1.15	0.027	0.141	0.128	0.057
*LHPP*	10q26.13	rs35837782	A	G	G	B-cell ALL	27694927	Vijayakrishnan J (2017)	1.00E-11	1.21 (1.15-1.28)	0.647	0.546	0.627	0.529
*ELK3*	12q23.1	rs4762284	A	T	T	B-cell ALL	27694927	Vijayakrishnan J (2017)	8.00E-09	1.19 (1.12-1.26)	0.456	**0.426**	0.300	**0.586**
*CEBPE*	14q11.2	rs2239630 *	A	G	A	ALL	29977016	Studd JB (2019)	1.66E-19	1.45	0.866	**0.542**	0.452	0.543
*CEBPE*	14q11.2	rs60820638	A	C	A	ALL	34750507	Jeon S (2021)	5.38E-08	1.19	0.717	0.719	0.704	**0.886**
*USP7*	16p13.2	rs74010351	A	G	G	T-cell ALL	30938820	Qian M (2019)	4.51E-08	1.44 (1.27-1.65)	0.180	0.099	0.067	0.029
*IKZF3*	17q21.1	rs2290400	T	C	T	ALL	29348612	Wiemels JL (2018)	2.05E-08	1.18 (1.11-1.25)	0.527	**0.562**	0.508	0.557
*IKZF3*	17q21.1	rs17607816	T	C	C	ALL	34750507	Jeon S (2021)	1.42E-07	2.11	0.004	0.005	0.028	0.000
*IGF2BP1*	17q21.32	rs10853104	C	T	T	B-cell ALL (*ETV6-RUNX1*)	31767839	Vijayakrishnan J (2019)	1.82E-08	1.33 (1.21-1.47)	0.666	0.489	0.507	0.329
*ERG*	21q22.2	rs8131436	G	C	C	ALL	31296947	de Smith AJ (2019)	8.76E-09	1.23 (1.16-1.31)	0.200	0.358	0.324	**0.471**

Risk allele frequency (RAF) from gnomAD v3; populations, African/African American (AFR), Latino/Admixed American (AMR), and non-Finnish Europeans (EUR).

P-values and odds ratios (OR) obtained from the studies indicated by PubMed ID, first author, and year of publication.

* Includes the putative causal variant where reported.

** Bolded where RAF in AMR is ≥5% higher than in EUR among gnomAD populations.

*** Based on 35 Indigenous American individuals in the Human Genome Diversity Project (HGDP). Bolded where this population has the highest RAF.

Most GWAS of childhood ALL have also been performed in individuals of predominantly European ancestry, although there have been several studies conducted in the United States that included Hispanic/Latino subjects in multi-ancestry analyses or that focused specifically on this population (60, 61, 64–67, 70). For several of the ALL-associated SNPs, including in *ARID5B*, *GATA3*, and *PIP4K2A* (70–73), the risk allele frequencies have been reported to be higher in Hispanic/Latino populations than in Europeans in reference population databases such as the Genome Aggregation Database (gnomAD) (**Table 1**), supporting a role for genetic variation in the higher incidence of ALL in Hispanics/Latinos. For example, the risk allele frequencies for the *ARID5B* SNP rs7090445, the *GATA3* SNP rs3824662, and the *PIP4K2A* SNP rs7088318 are 44%, 29%, and 70% in Latino/Admixed American populations versus only 32%, 18%, and 60% in non-Finnish European populations, respectively, as reported in gnomAD (v3.1) (74) (**Table 1**). *ARID5B* and *GATA3* SNPs have also been associated with an increased risk of relapse in childhood ALL patients (59, 71, 75), supporting that genetic variation in these genes also contributes to the inferior outcomes of Hispanic/Latino patients.

The *GATA3* risk locus appears to contribute specifically to the increased prevalence of the high-risk Ph-like subtype of ALL in Hispanic/Latino patients (59) – the noncoding *GATA3* variant rs3824662 was associated with minimal residual disease after induction therapy (75) and was recently demonstrated to have a functional role in the development of *CRLF2* rearrangements that frequently drive the Ph-like ALL phenotype (76). More specifically, the rs3824662 risk allele increases the expression of *GATA3*, which binds to the promoter of *CRLF2* and appears to induce looping of this locus on chromosome X to a downstream super-enhancer at *P2RY8*, resulting in the chromatin region between these two loci becoming more open and susceptible to rearrangements (76). The Ph-like subtype and *CRLF2* rearrangements have been found to be more prevalent in AYA and older adult ALL patients than in childhood ALL (77), and in particular among older Hispanic/Latino patients (78, 79). For example, in a study of adult patients with Ph-like ALL, 68% were Hispanic/Latino and 23% were non-Hispanic White, whereas among B-other ALL patients only 30% were Hispanic/Latino and 51% were non-Hispanic White (78). Adults appear to be more susceptible to developing Ph-like ALL than children, and the *GATA3* risk SNP rs3824662 may be a major factor underlying the ~2-fold greater risk of ALL in Hispanic/Latino adults than in non-Hispanic Whites (8). Another interesting aspect is that a higher percentage of Hispanic/Latino ALL patients than non-Hispanic White patients are diagnosed over the age of 10, an age group where the Ph-like phenotype is also more common (20).

In addition to the higher risk allele frequencies of *ARID5B*, *GATA3*, and *PIP4K2A* SNPs in Hispanic/Latino populations, their risk alleles are positively correlated with proportions of Indigenous American ancestry among Hispanic/Latino individuals (71–73). Furthermore, genetic variation at the *ERG* gene was found to be significantly associated with childhood ALL risk in Hispanics/Latinos but not in non-Hispanic Whites and, among Hispanics/Latinos, the *ERG* risk alleles were associated with Indigenous American ancestry and the effects of this locus on ALL risk were larger in individuals with increased Indigenous American ancestry (64, 65). Results from the analyses of individual ALL-associated SNPs, therefore, suggest that Hispanics/Latinos may harbor more risk alleles on average than individuals of European ancestry. To systematically study this, we recently calculated a polygenic risk score (PRS) to aggregate the effects of the known ALL GWAS SNPs. This revealed that the ALL PRS was on average significantly higher in Hispanics/Latinos than in non-Hispanic Whites, in both cases and controls separately (66).

It is possible that additional genetic loci, or variants within known loci, contribute to the increased ALL risk in Hispanics/Latinos. Indeed, we recently conducted a fine-mapping analysis across the *IKZF1* gene at chromosome 7p12.2 and discovered a novel association signal, with a lead SNP rs76880433 that is relatively common in Hispanic/Latino populations (17.6%) but almost absent in non-Finnish Europeans (0.2%) in gnomAD (v3.1), and that confers an effect size of ~1.44 per risk allele (80). Indeed, Hispanic/Latino populations appear to be unique in harboring three independent risk loci at the *IKZF1* region. Similar to previous findings for ALL risk SNPs in *ARID5B*, *GATA3*, *PIP4K2A*, and *ERG*, the *IKZF1* SNP rs76880433 risk allele was positively associated with global and local Indigenous American ancestry. This newly discovered association locus at *IKZF1* appears to explain a substantial portion of the increased ALL risk in Hispanics/Latinos compared to non-Hispanic Whites. There are likely additional as yet undiscovered risk variants that contribute to this disparity in ALL incidence, which may require studies with larger sample sizes to detect variants with smaller effects or sequencing studies to identify variants that may have been missed by standard GWAS approaches (for example, structural variants or SNPs in repetitive genomic regions).

## Genetic variation and ALL risk in children in Latin America

Genome-wide genetic studies of ALL in Hispanic/Latino children have largely been confined to the United States. These studies have revealed novel insights into the genetic architecture of ALL and discovered genetic loci that appear to contribute to the increased ALL risk in Hispanics/Latinos; however, the composition of the Hispanic/Latino population in the United States does not fully represent the diversity of populations in Latin America, in terms of country of origin and, thus, genetic ancestry. For example, according to the 2022 Census Bureau, people of Mexican origin comprised ~61% of the ~63 million Hispanics/Latinos living in the United States, followed by Puerto Rico (~9%) and Cuba (~4%) as the other main places of origin (81). This is clearly distinct from the constitution of populations across Latin America itself, where Brazil contributes the largest number of individuals. Thus, the relevance of findings from genetic studies conducted in Hispanics/Latinos in the United States will vary depending on the Latin American country in question, especially given the regional variability in the relative proportions of Indigenous American, European, and African ancestries, as discussed earlier.

The findings from United States-based studies of Hispanics/Latinos are clearly of high relevance in Mexico, and a previous study of 285 childhood ALL cases and 476 controls in the Mexican Interinstitutional Group for the Identification of the Causes of Childhood Leukemia (MIGICCL) confirmed the association of *ARID5B* SNPs with ALL risk in the Mexican population (82). In another study in MIGICCL, a gene-environment interaction analysis identified a possible interaction between a variant in the xenobiotic metabolism gene *NAT2* and certain exposures including fertilizers and an increased childhood ALL risk (83). SNP array genotyping has been performed in a study of almost 200 childhood ALL patients from Guatemala, in which the majority of individuals have high (>75%) proportions of Indigenous American ancestry. In this patient cohort, Indigenous American ancestry was positively associated with the frequency of somatic *CRLF2* rearrangements and with an increased risk of relapse (50). In a case-control study of childhood ALL in Brazil, including 121 cases and 155 controls, genotyping of a small number of candidate SNPs as well as ancestry informative markers was performed. An association with ALL risk SNPs in *ARID5B* and *CEBPE* was confirmed, and as might be predicted the Brazilian childhood ALL cases had significantly higher Indigenous American ancestry proportions than controls (34% vs. 28%) (84). These few examples notwithstanding, genetic association studies of childhood ALL in Latin America are lacking. Additional studies are warranted to investigate the effects of known ALL risk variants in children across Latin America and potentially discover novel risk loci, and to incorporate germline genetic information in studies of environmental risk factors in epidemiological studies of childhood ALL, for example in the MIGICCL.

**Figure 2:**
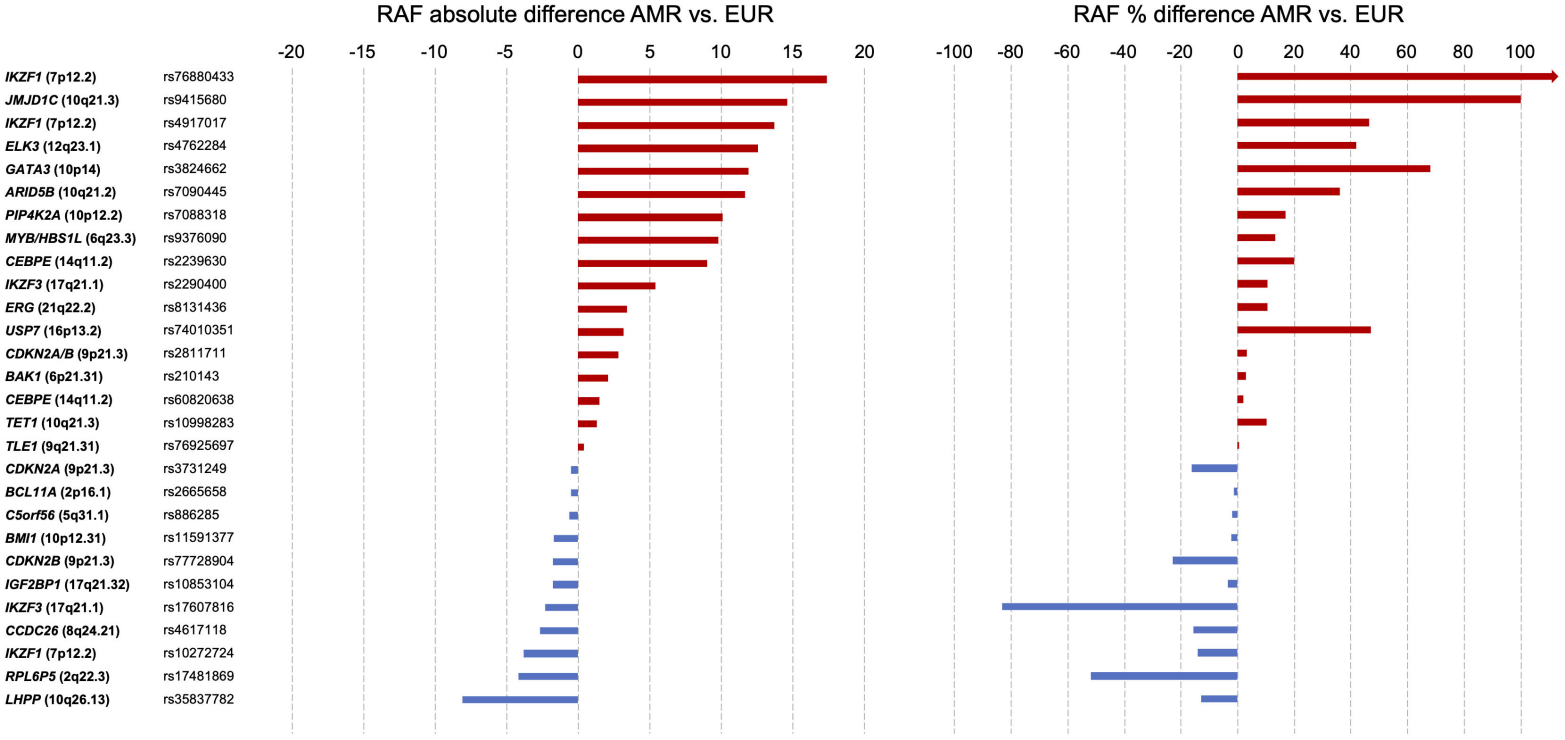
Risk allele frequency of selected SNPs associated with childhood acute lymphoblastic leukemia risk. Single nucleotide polymorphisms (SNPs, n=28, **Table 1**) are grouped by nearest genes in each panel. Left: Absolute difference in risk allele frequency between Latinos/Admixed Americans and non-Finnish Europeans in gnomAD v3.1. Right: Percentage difference in SNP risk allele frequencies between Latinos/Admixed Americans and non-Finnish Europeans. Percentage change equation: {[(Risk allele frequency of Latinos/Admixed Americans)/(Risk allele frequency of non-Finnish Europeans)] - 1} x 100. Horizontal bars are colored by the direction of percentage difference.

As discussed earlier, GWAS of childhood ALL have identified several risk loci that are associated with Indigenous American ancestry and have demonstrable differences in risk allele frequency and/or in effect size across population groups. In **Table 1**, we have summarized the childhood ALL-associated SNPs previously reported by GWAS. We obtained the risk allele frequencies of each SNP reported in Latino/Admixed American, non-Finnish European, and African American populations in gnomAD (v3.1). For more than one-third (10/28) of SNPs, the risk allele frequency in Latinos/Admixed Americans was at least 5% higher than in Europeans, including the aforementioned SNPs in *ARID5B*, *GATA3*, and *PIP4K2A*, as well as two SNPs in *IKZF1*, and one variant each in *CEBPE*, *MYB*/*HBS1L*, *JMJD1C*, *ELK3*, and *IKZF3* (**Table 1**). In addition to comparing the absolute differences in risk allele frequencies, we calculated the percent difference in risk allele frequency between Latino/Admixed American and European populations (**Figure 2**). There are 13/28 SNPs with a risk allele frequency at least 10% higher in Latinos/Admixed Americans than Europeans versus only 7 SNPs with a frequency >10% higher in Europeans. Notably, the *ARID5B* SNP rs7090445 and *GATA3* SNP rs3824662 risk allele frequencies are 36% and 68% higher in Latinos/Admixed Americans than in Europeans, respectively. In addition, the risk allele frequencies of two SNPs at *IKZF1*, including the newly discovered independent association signal at this locus, have markedly higher risk allele frequencies in Latinos/Admixed Americans than Europeans (**Figure 2**).

We also examined the risk allele frequencies of childhood ALL-associated SNPs in Indigenous American individuals from the Human Genome Diversity Project (HGDP) (85). Although based on a small sample size of 35 individuals, it is interesting to note that for several of the loci the risk allele frequencies are highest in Indigenous Americans compared to Latinos/Admixed Americans and other populations, including at *ARID5B*, *GATA3*, *CEBPE*, *IKZF1*, and *ERG* (**Table 1**). This has further implications for childhood ALL risk in countries across Latin America, particularly those with highly indigenous populations.

## Conclusions and future directions

In this review, we discuss the role of genetic variation in the increased risk of childhood ALL in Hispanics/Latinos compared to non-Hispanic individuals, and the implications for children living in Latin America. Based on the research to date, it appears that children of Hispanic/Latino ethnicity both in the United States and in Latin America, and particularly those who harbor greater proportions of Indigenous American ancestry in their genomes, are more likely to carry a larger number of genetic risk alleles that predispose to the development of ALL. There are, however, several unanswered questions that warrant further research.

In terms of genetic risk, the extent to which common variants account for the increased ALL risk in Hispanics/Latinos has not been formally tested. This is true both for the individual variants that have higher risk allele frequencies in Hispanic/Latino populations, for example in *ARID5B*, *GATA3*, and *IKZF1*, and for the combined effects of all known ALL risk SNPs. Furthermore, the question of why there appears to be a higher frequency of ALL risk alleles in Hispanics/Latinos remains unanswered. It is possible that these variants conferred some evolutionary advantage in Indigenous American populations, perhaps following European colonization of the Americas which led to the decimation of indigenous populations in part because of a lack of immunity to the pathogens brought over by Europeans (86–88), and analysis of signals of selection at ALL risk loci is warranted.

Specific to children in Latin America, how do the effect sizes of ALL risk SNPs compare to those determined from GWAS of childhood ALL conducted in Hispanics/Latinos in the United States, given the potential differences in environmental exposures between countries? To answer this will require genome-wide SNP genotyping or sequencing of childhood ALL cases and controls from countries across Latin America. This is already under way for Mexican ALL cases and controls included in the MIGICCL study, and similar efforts in other countries are needed. Furthermore, it will be important to understand the genetic diversity that exists across Latin American populations as well as Indigenous American subpopulations (*e.g.*, in Mexico there are at least 60 Indigenous American groups) (51, 89–91) and, in turn, how different Indigenous American ancestries might influence allele frequencies and effect estimates at childhood ALL risk loci. Moreover, it is essential to consider the heterogeneity of Hispanic/Latino populations in epidemiological studies that investigate the racial/ethnic disparities in ALL incidences.

In addition, sequencing studies in familial and sporadic ALL patients have discovered rare pathogenic germline variants in genes encoding hematopoietic transcription factors including *IKZF1*, *PAX5*, and *ETV6* (5, 92–96); however, the frequency of such rare variants in ALL predisposition genes across different racial/ethnic groups of patients, and hence their potential role in the increased risk of ALL in Hispanics/Latinos, has not been addressed. This would require sequencing studies in large numbers of childhood ALL cases across different Hispanic/Latino populations in the Americas, which may also discover population-specific founder mutations that predispose to ALL similar to the Brazilian founder mutation in *TP53* (97).

Although we focus on genetic risk in this review, there are likely to be environmental factors that contribute to the differences in ALL incidence rates between populations. Indeed, recently discovered risk factors including cytomegalovirus infection at birth and Cesarean section were found to confer a greater effect on ALL risk in Hispanics/Latinos than in non-Hispanic Whites (98, 99). In contrast, the reported protective effect of early-life exposure to common infections on childhood ALL risk, which supports the Greaves’ “delayed infection” hypothesis (4, 100), appears to be reduced in Hispanics/Latinos (101, 102). This could perhaps be a consequence of the fact that infectious agents are more common in Hispanic/Latino populations. Overall, little is known regarding the contribution of environmental exposures to the disparity in ALL risk in Hispanics/Latinos, or how genetic variants may interact with environmental exposures in the development of ALL.

It is important to highlight that in recent decades the incidence of childhood ALL (and of ALL in AYAs and older adults) has been increasing significantly in Hispanics/Latinos but not in other racial/ethnic groups in the United States (8, 15), and this certainly warrants further investigation. Given that the frequencies of ALL risk alleles are unlikely to have changed over such a short period of time, there may be environmental exposures or non-genetic factors that have been on the rise recently and that may confer risk of ALL. One such factor may be childhood obesity, which has increased in incidence in the United States in recent years (103), has a higher prevalence among Hispanic/Latino children (104, 105), and has been associated with an increased risk of ALL in Hispanics/Latinos (106) and with an increased frequency of somatic *CRLF2* rearrangements among Hispanic/Latino ALL patients (107). In Mexico, the incidence of ALL has been reported to have remained stable over recent years, although the analysis was limited to patients treated at public hospitals. We acknowledge that high-quality cancer registry data have been lacking for several countries in Latin America (108), although progress has been made in recent years (109). The lack of reliable population-based data may impact the comparison of reported ALL incidence rates between countries as well as the reported trends in ALL incidence over time; however, the acute nature of childhood ALL means it is unlikely to be over diagnosed and the relatively high incidence reported in countries such as Mexico may actually be an underestimation. Further research on the trends in ALL incidence rates across countries in Latin America is needed.

Accessibility to health systems may also be linked to the trend in ALL incidences and patient outcomes in Latin American countries (110, 111). In Mexico, the government program known as Seguro Popular (Popular Insurance) that aimed to provide universal healthcare has recently disappeared, leaving children with cancer without the possibility of free medical care (110). The possibility that the poorest populations, where there is a greater prevalence of indigenous ancestry, do not go to highly specialized hospitals for medical care because they do not have the economic resources to pay for it could lead to changes in the reported incidence of these diseases. Because of the rarity of this disease, the underreporting of even a small number of patients may lead to a failure to detect an increasing trend in ALL incidence over time. Unfortunately, this situation may exist in several Latin American countries.

It is important to keep in mind that ALL remains a relatively rare disease in children, including in Hispanics/Latinos, and that the lifetime risk of developing ALL even among children with high Indigenous American ancestry would remain relatively low. However, elucidating the causes of the increased risk of ALL in Hispanic/Latino children remains a research priority, both in the United States and for countries across Latin America. Characterizing the genetic risk associated with childhood ALL could improve our understanding of the disparities in ALL incidence across populations. Finally, identifying children with a higher risk of developing ALL, via genetic screening and exposure analysis, will be important for future precision prevention efforts that aim to alleviate the disparity in childhood ALL incidence in Hispanics/Latinos.

## Author contributions

AdS: Writing – original draft, Writing – review & editing. SJ-M: Writing – original draft, Writing – review & editing. JM-A: Writing – original draft, Writing – review & editing.
